# Computational fluid dynamics simulation of two-phase flow patterns in a serpentine microfluidic device

**DOI:** 10.1038/s41598-023-36672-6

**Published:** 2023-06-10

**Authors:** Younes Amini, Valiyollah Ghazanfari, Mehran Heydari, Mohammad Mahdi Shadman, A. Gh. Khamseh, Mohammad Hassan Khani, Amin Hassanvand

**Affiliations:** 1 Nuclear Fuel Cycle Research School, Nuclear Science and Technology Research Institute, Tehran, Iran; 2 Department of Polymer Engineering, Faculty of Engineering, Lorestan University, Khorramabad, Iran

**Keywords:** Energy science and technology, Engineering

## Abstract

In the current research work, the flow behavior of a liquid–liquid extraction (LLE) process in a serpentine microchannel was analyzed. The simulation was performed using a 3D model and the results were found to be consistent with experimental data. The impact of the flow of chloroform and water on the flow model was also examined. The data indicate that once the aqua and organic phases flow rates are low and similar, a slug flow pattern is observed. However, as the overall flow rate raises, the slug flow transforms into parallel plug flow or droplet flow. An increment in the aqua flows while maintaining a constant organic phase flow rate results in a transition from slug flow to either droplet flow or plug flow. Finally, the patterns of flow rate in the serpentine micro-channel were characterized and depicted. The results of this study will provide valuable insights into the behavior of two-phase flow patterns in serpentine microfluidic devices. This information can be used to optimize the design of microfluidic devices for various applications. Furthermore, the study will demonstrate the applicability of CFD simulation in investigating the behavior of fluids in microfluidic devices, which can be a cost-effective and efficient alternative to experimental studies.

## Introduction

The use of two-phase liquid–liquid (LL) systems is prevalent in chemical treatment, for instance, polymerization, nitration, chlorination, and reactive and solvent extraction^1–5^. Those procedures are mostly hampered by transport limitations, such as small mass transfer rates^6–8^. To overcome these limitations, miniaturization has been recognized as a promising method of process intensification, by reducing transport resistance and increasing transport rates^9–11^. The utilization of micro-spaces in devices can result in high heat and mass transfer rates^12–17^. The higher interfacial zone-to-volume fraction in micro-scale binary schemes compared to macro-scale systems results in enhanced heat and mass transfer rates and increased process efficiency, which can be higher by an order of magnitude compared to conventional systems. Additionally, the ease of scale-up developed safety, and reduced inventory requirements, specifically for systems using risky and exclusive chemicals, make microfluidic devices appropriate for a broad range of applications. The effectiveness of a specific system in LL microchannels depends greatly on the flow schemes of the two non-miscible liquids^18–21^.

Microfluidic flow patterns refer to the behavior of the fluid in microscale channels or devices. Three main flow, parallel, droplet and slug flow, occurs in microfluidic systems. Flow maps show graphically of these main flows versus the flow rate of two phases. Understanding microfluidic flow patterns is important for designing and optimizing microfluidic devices for specific applications. By controlling the flow pattern, researchers can manipulate the behavior of fluids in microscale channels and develop devices that can perform precise chemical reactions, separations, and detections^22–24^.

Several LL flow patterns were scrutinized in microfluidic tools based on factors such as micro-channel size and shape, physical characteristics of the liquids (for instance viscosity and surface tension), flow rate, the flow ratio of the liquids, and the wetting behavior of the micro-channel walls^25–27^. The maximum usual LL flow patterns in two-phase micro-channels include slug flow, plug flow, and droplet flow. Slug flow is favored for numerous systems due to the interior rotation inside the slugs of two phases and the diffusion among the contiguous slugs. Nonetheless, comprehensive phase split-up inside the micro-fluidic tool remains a challenge in slug flow regimes. Slug hydrodynamics, such as slug length and speed, is of significant significance as they affect the performance of microfluidic devices^28–31^.

The researchers have put forward various scaling laws to estimate the length of the slug in a two-phase liquid–liquid flow^32–35^. It has been noted in previous studies that the speed of the slug is directly related to the overall speed of the fluid^36–38^. In plug flow, separating the liquid into two phases is possible on a chip because of the creation of a stable LL interface down the center of the micro-channel, which could be achieved by using a Y-split at the microchannels exit . However, the method of transport in plug flow is limited to diffusion, leading to low system effectiveness compared to processes that utilize segmented flow^39–41^. Several research projects were performed to create flow regime maps^42–44^. These maps could be sketched according to the flow rate of binary systems, but they cannot be used as a general representation as they do not take into account all factors that impact the flow sketches. Therefore, researchers have suggested dimensionless quantities, such as Re, We, and Ca, to create general flow configuration maps^45^. Various mixtures of these dimensionless quantities were utilized as coordinates for generalized flow diagrams. Waelchli et al.^46^ employed the Buckingham Pi-theorem to find overall flow behavior for gas–liquid flows and proposed an association of Reynolds (Re) and Weber (We) numbers to generalize experimental data. Meanwhile, Cao et al.^36^ examined the LL flow schemes in a non-circular glass micro-channel and created comprehensive flow model transitions according to the force analysis. It has also been suggested to utilize Re and We numbers to estimate flow model transitions. Yagodinitsyna et al.^47^ employed an easy dimensionless analysis for LL flow to find a universal factor for flow pattern maps. They proposed the We number multiplied by the Ohnesorge number (Oh) as a novel factor to simplify the flow models of their analyzed methods. The units of physical variables used in the dimensionless analysis and The dimensionless numbers are in Tables 1 and 2.

**Table 1:** The units of physical variables used in the dimensionless analysis.

Physical variables	Dimensions
u (ms^−1^)	LT^−1^
D_h_ (m)	L
$\rho \;({\text{kg}}\;{\text{m}}^{ - 3} )$	ML^−3^
$\mu \;({\text{Pa}}\;{\text{s}})$	ML^−1^ T^−1^
$\gamma \;({\text{Nm}}^{ - 1} )$	MT^−2^

**Table 2:** The dimensionless numbers.

Dimensionless numbers	Formula
Renolds number	$Re=\frac{\rho ud}{\mu }$
Capillary number	$Ca=\mu u/\sigma$
Weber number	$We=\frac{\rho {u}^{2}d}{\sigma }$
Ohnesorge number	$Oh=\frac{\mu }{\sqrt{\rho \sigma L}}=\frac{\sqrt{We}}{Re}$

Darekar et al.^43^ investigated the flow models of standard removal schemes in Y-junction micro-channels and assessed the effectiveness of using Re, Capillary (Ca), We, and Weber multiplied by (We Oh) numbers for general flow map report. The last two quantities provided the best outcomes. It is crucial to conduct a dimensionless study to determine the appropriate grouping of dimensionless quantities to create a general flow diagram. Despite being assumed a guaranteeing alternative to traditional methods, the low output of a particular micro-channel remains a challenge, requiring the scaling-up of microfluidic devices for industrial manufacture. An answer to this issue is to develop multiple micro-channels in parallel, increasing the output while avoiding complications of traditional scaling methods^48^. To the best of our knowledge, there is little information on the flow model of two non-miscible mixtures in a parallel microfluidic tool, with the majority of earlier studies examining parallelized drop-based microfluidic tools for creating even emulsion drops^49–53^. Kassid et al.^54^ examined the flow dissemination of a LL scheme in six distinct capillaries to evaluate the mass transfer efficiency of the kerosene (+ acetic acid)—water scheme.

This study used CFD to determine the flow map in a serpentine microchannel. three regime flow in micro-channel, droplet, slug, and plug flow, were developed. This study focuses on the micro-channel to reveal their flow map at various flow rates. Furthermore, the multi-phase flow interior of the micro-channel was calculated mathematically to distinguish the flow model in the serpentine micro-channel. The outcomes of mathematical simulations were proved via experimental results. The received data from the simulations were compatible with the experimental results, indicating that the mathematical simulations accurately modeled the flow behavior in the microchannel..

## Mathematical simulation

### Governing correlations

The calculations for multiphase flows involving LL interfaces are done using the volume of fluid (VOF) technique^55–57^. This method is a popular technique used in CFD simulations to model the behavior of multiphase flows. The VOF method is a Eulerian method that tracks the interface between two immiscible fluids by solving the Navier–Stokes equations for each fluid phase while tracking the volume fraction of each phase in each computational cell.

The VOF method works by dividing the computational domain into a grid of small cells, and at each cell, the volume fraction of each fluid phase is tracked. The interface between the two fluids is defined as the region where the volume fraction of a phase changes from 0 to 1 or vice versa. This model is effective in monitoring the boundary between two phases that do not mix. In the VOF system, parameters such as pressure and speed are common to binary phases and reflect the average volume amounts. The correlations for conserving the mass and momentum of two incompressible, non-miscible liquids are volume-averaged and can be found in^58–65^:

1 $$\frac{\partial }{\partial t}\left(\rho \right)+\nabla .\left(\rho u\right)= 0$$

2 $$\frac{\partial }{\partial t}\left(\rho {u}_{j}\right)+\frac{\partial }{\partial {x}_{i}}\left(\rho {u}_{i}{u}_{j}\right)= -\frac{\partial P}{\partial {x}_{j}}+\frac{\partial }{\partial {x}_{i}}\mu \left(\frac{\partial {u}_{i}}{\partial {x}_{j}}+\frac{\partial {u}_{j}}{\partial {x}_{i}}\right)+\rho {g}_{i}+{f}_{\sigma }$$

In these correlations *ρ*, *μ*, *u, ρg*_*i*_, and *f*_*σ*_ are the density of the fluid, the dynamic viscosity, the speed vector, the gravity force, and the exterior body forces, correspondingly.

The density of the mixture and viscosity is estimated via volume fraction averaging^66–70^.

3 $$\rho ={{\alpha }_{1}\rho }_{1}+{{\alpha }_{2}\rho }_{2}$$

4 $$\mu ={{\alpha }_{1}\mu }_{1}+{{\alpha }_{2}\mu }_{2}$$

where $\mathrm{\alpha }$ is the volume percentage and digits belong to the phase. It is evident that the sum of the volume percentage of the specific phase is supposed to be hundred percent, as follows

5 $$\sum_{i=1}^{n}{\alpha }_{i}=1$$

The volume fraction in the interface ranges from 0 to 1, making it crucial to accurately track the interface within cells.

To do this, it is necessary to solve a continuity correlation for the volume percentage of unity or multiple phases. The continuity equation for the volume fraction of the i-th phase is expressed as follows:

6 $$\frac{\partial }{\partial t}\left({\alpha }_{i}{\rho }_{i}\right)+\nabla .\left({\alpha }_{i}{\rho }_{i}{\overrightarrow{\nu }}_{i}\right)=0$$

At the gas–liquid interface, a pressure jump occurs due to the surface tension difference between both sides. This difference is considered in balance and its slope should match the added body force in the momentum balance. The pressure jump disjunction is estimated as described in^66,71,72^:

7 $${f}_{\sigma }=-\sigma \left(\nabla .\left(\frac{\nabla \alpha }{\left|\nabla \alpha \right|}\right)\right)\left(\nabla \alpha \right)$$

The wetting characteristics of the substance are analyzed by examining the water droplet contact angles on the glass side. The contact angles are measured using a standard goniometer. The physical Characteristics of the fluids are displayed in Table 3.

**Table 3:** Characteristics of fluids^45^.

Fluid	${\rho } \left(\frac{kg}{{m}^{3}}\right)$	${\mu }$ $\left(cP\right)$
VWater	998.2	1
Cloroform	1489	0.58
	surface tension (N/m)	0.0315

### Mathematical method

#### Geometry and Grid

**Figure 1:**
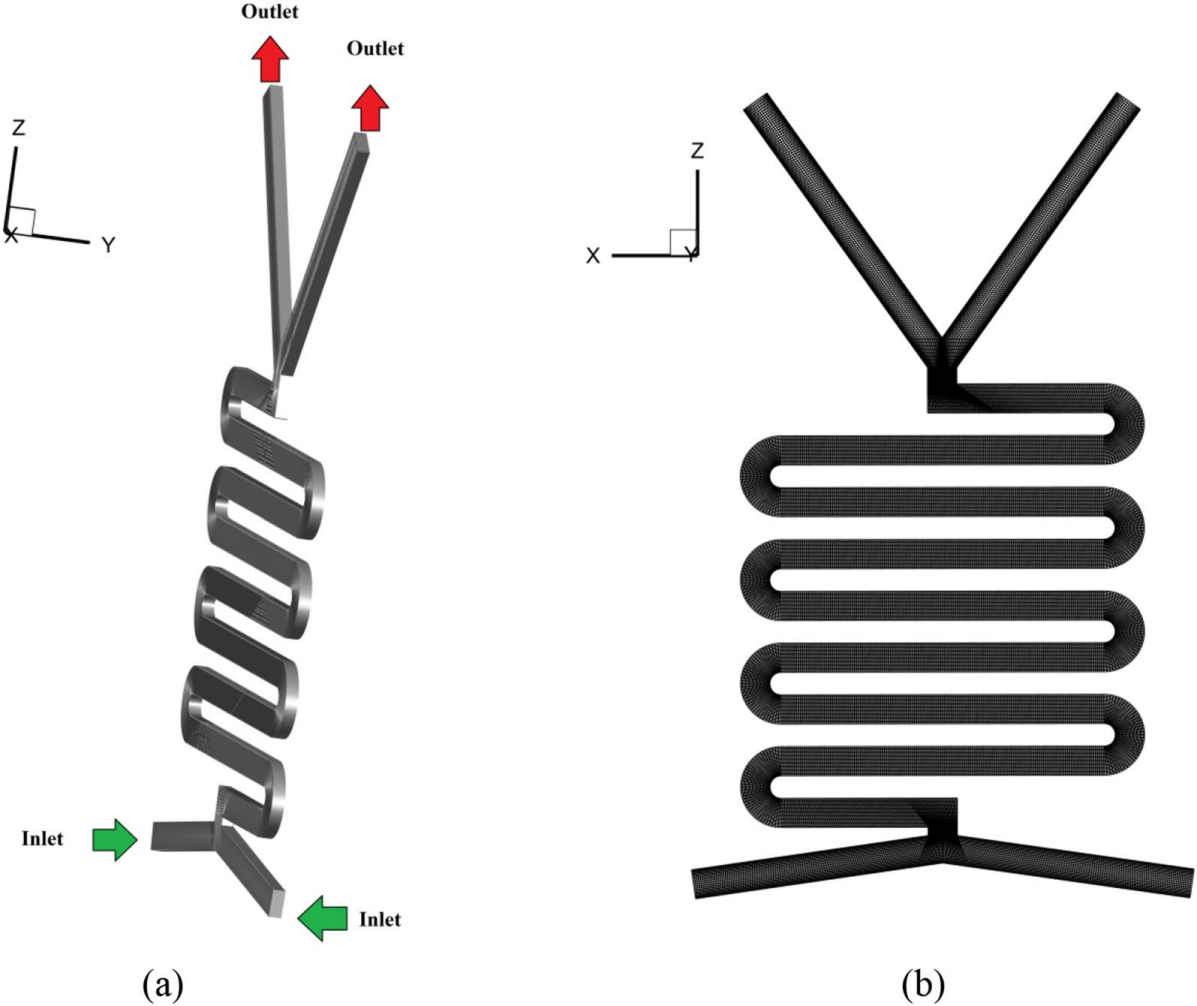
(**a**) Geometry of micro-channel (**b**) creating the grid.

In this study, three-dimensional micro-channel geometry was chosen for the flow map analysis. The geometry and mesh are shown in Fig. 1. The microchannel has a smooth profile with a surface roughness average of 0.22 μm. The area of the cross-section is approximately 0.13 mm^2^ with a hydraulic diameter of 0.32 mm. The length of each microchannel (mixing part) is 102 mm. Three-dimensional structured elements were used for mesh generation due to their ability to handle complex geometries and reduce errors. The computational domain was initially established using components with standard side lengths of 5, 3, 2, and 1 µm (mesh numbers of 495,000, 950,000, 1,920,000, and 3,850,000). After explaining the flow field and volume percentage correlations, the slug length of the simulation was compared to the experimental data and plotted. The outcomes are presented in Table 4, with comparative errors in the estimation of the slug length for the 1 and 2 µm elements being less than 1%. Thus, networks with average lengths of 2 µm were chosen as the threshold for the numerical simulation.

**Table 4:** Impact of mesh dimension on the slug length.

First component size	1 µm	2 µm	3 µm	5 µm
Slug lenght	2.80	2.83	2.98	3.41

In order to assess the quality of the mesh used in the simulation, the aspect ratio and skewness of the mesh elements were analyzed. The aspect ratio measures the elongation of each mesh element, and a high aspect ratio can result in inaccurate simulations. The skewness measures the deviation from a regular shape, and a high skewness can lead to numerical instabilities. The results of the analysis showed that the aspect ratio of most elements was below 3, indicating that the mesh was not excessively elongated. Additionally, the skewness was within the acceptable range of less than 0.5. Based on these results, it was concluded that the mesh quality was acceptable and suitable for use in the simulation.

#### Mathematical simulation

For the two-phase flow simulations, the uniform inlet velocity boundary conditions for both liquid phases are implemented. At the outlet, it is assumed that the Pressure outlet boundary condition for liquid and gas. At the walls, a no-slip boundary condition for liquid phases is imposed. The finite volume method was used for the numerical solutions. The SIMPLE algorithm was selected to compute the pressure–velocity coupling. The second-order upwind discretization was used for momentum. The convergence criterion of the residual errors was set to 10^–4^. For this purpose, the finite volume method of the ANSYS FLUENT software package was used to obtain numerical solutions.

## Results and discussions

**Figure 2:**
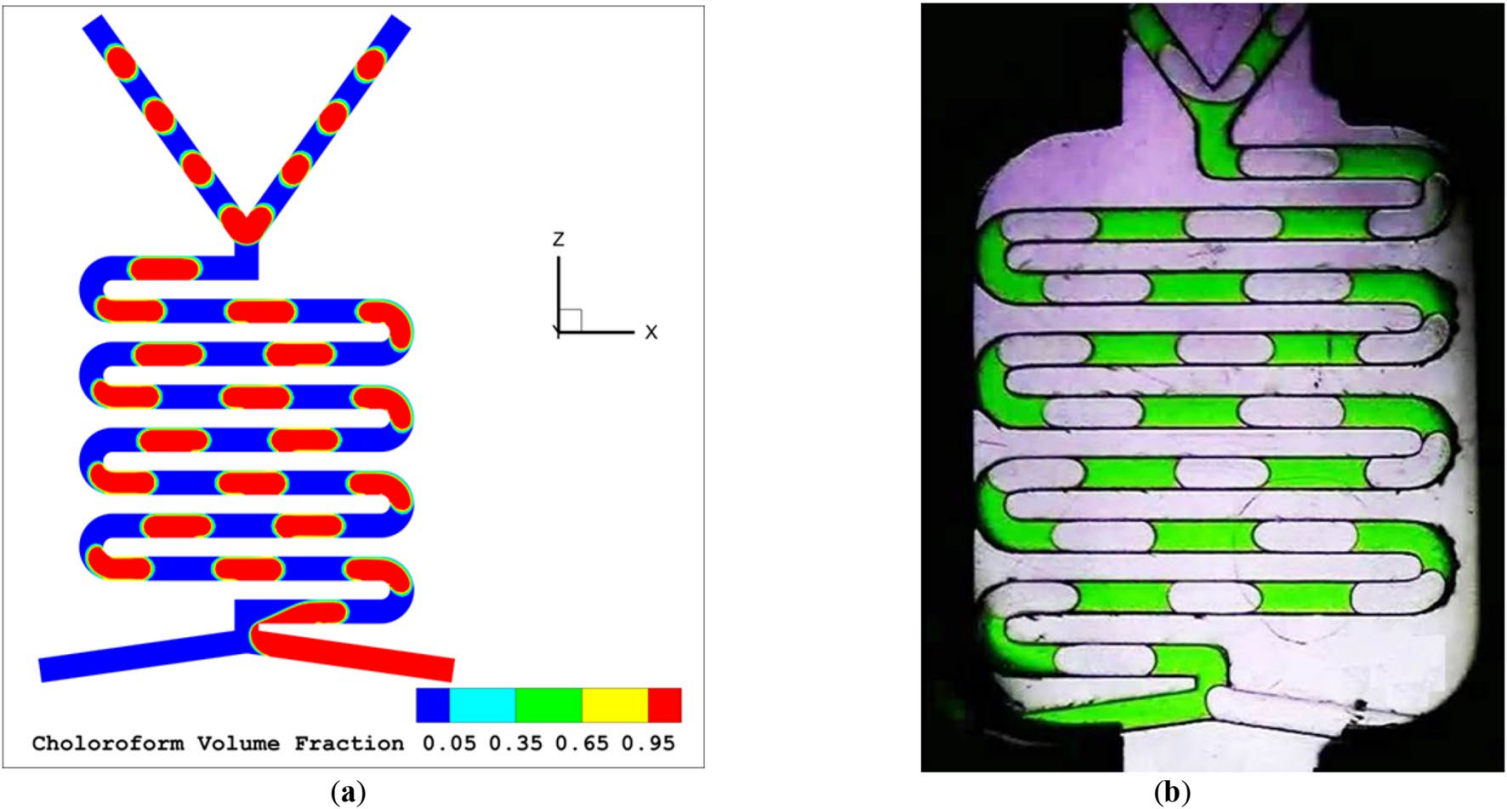
The slug flow regime (**a**) simulation (**b**) experimental work^45^.

**Figure 3:**
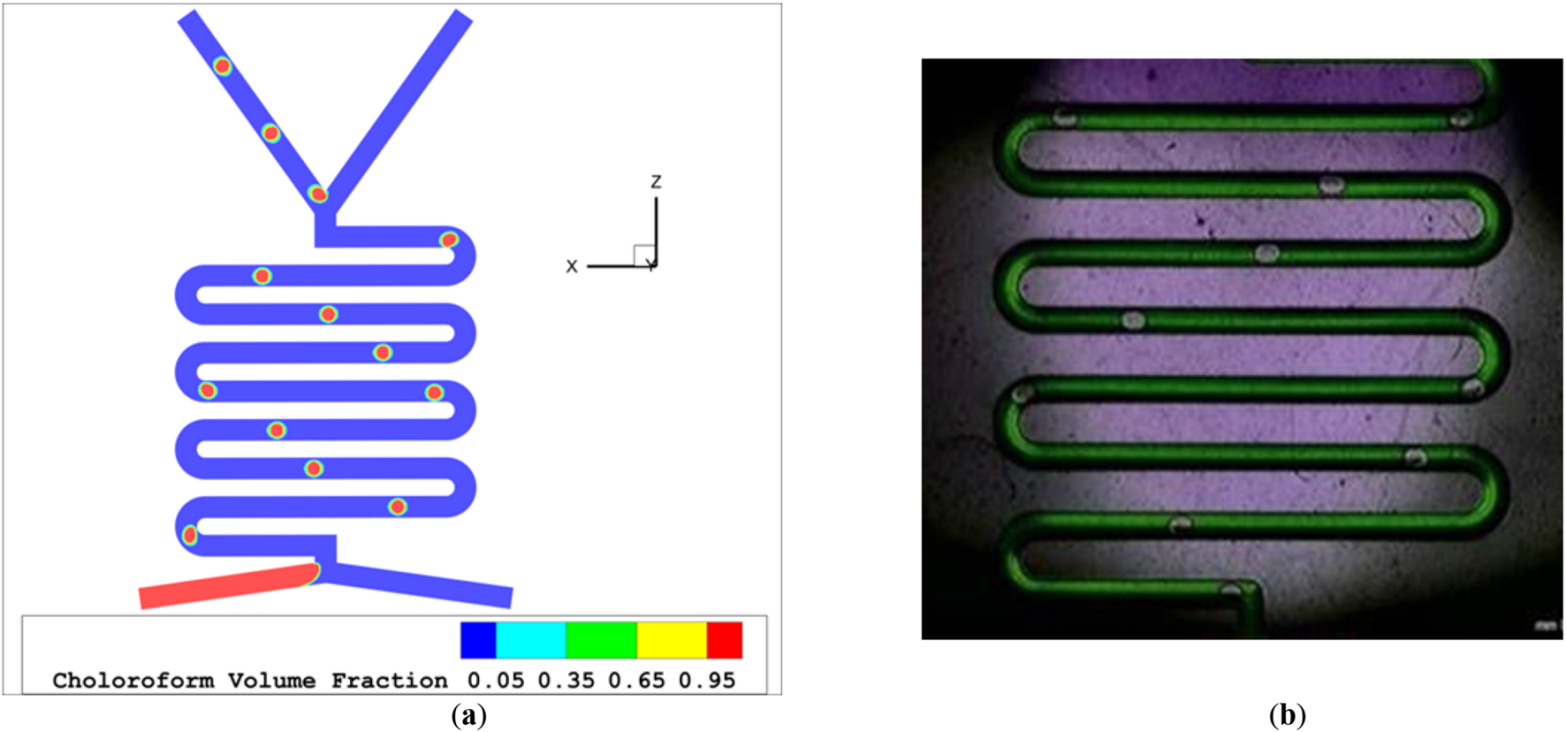
The droplet flow regime (**a**) simulation (**b**) experimental work^45^.

**Figure 4:**
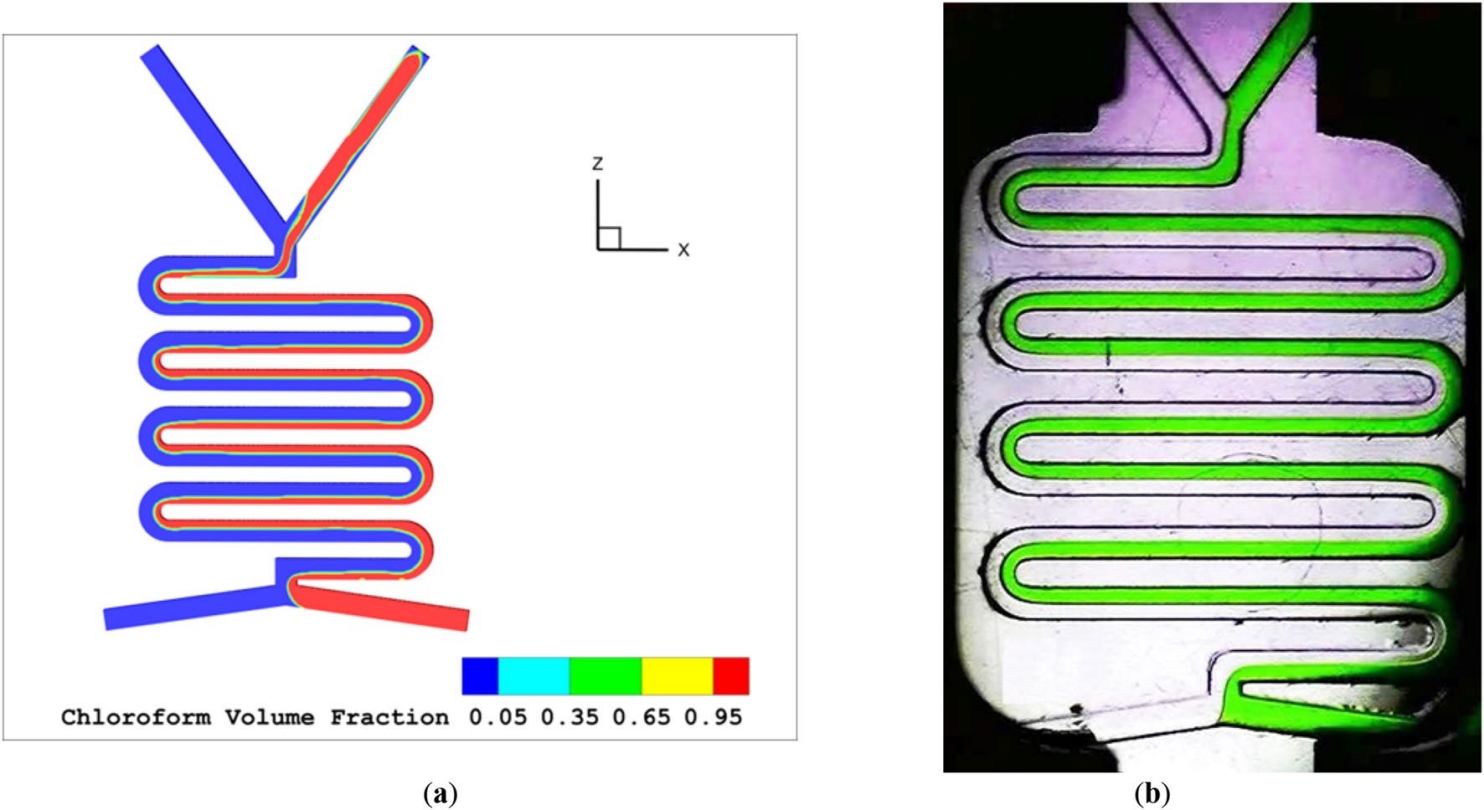
The plug flow regime (**a**) simulation (**b**) experimental work^45^.

**Figure 5:**
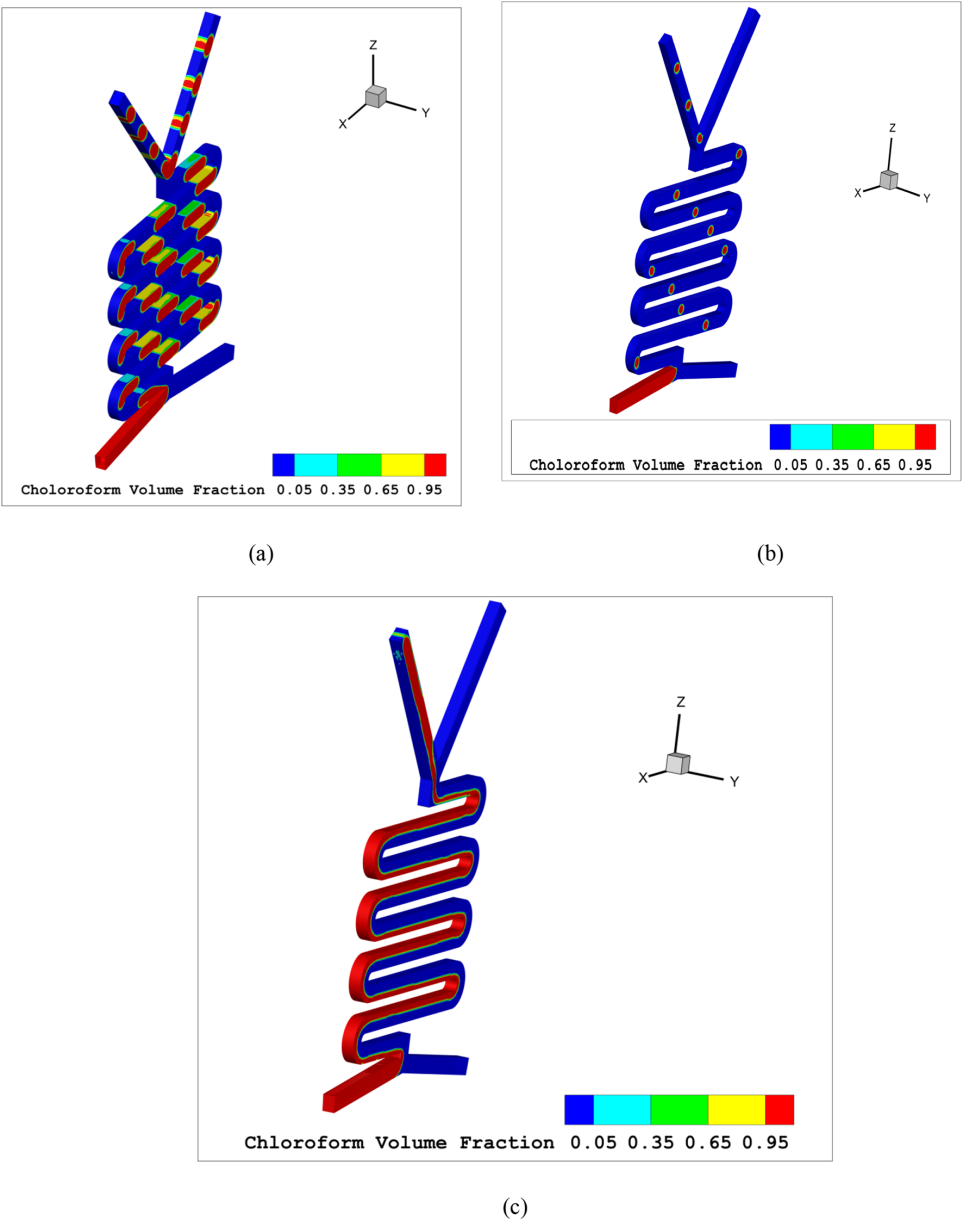
3D chloroform volume fraction (**a**) slug flow (**b**) droplet flow (**c**) parallel flow.

The diagrams that depict the flow models of different LL schemes, known as flow maps, demonstrate how the flow rate affects the flow regime. Figures 2, 3, 4 and 5 present the different flow regimes—slug flow, droplet flow and plug flow—in a serpentine microfluidic channel as validated by the experimental findings of Asadi et al.^45^. The simulation outcomes match well with the experimental work.

Figure 2 illustrates the occurrence of slug flow once the flow rates of the aqua and organic phases are somewhat small and similar. In this figure, the flow rates are 100 and 100 µl per minute for the aqua and organic phases, respectively. As depicted, the organic phase enters the main channel first and takes up a large portion of its cross-section, causing the continuous phase to be blocked to a significant extent. These results in increased drag force being applied to the interface, leading to the gradual and complete entry of the organic phase into the major channel over time. The pressure gradient created in the forming clump and the drag force acting on the interface counteract the surface tension force, causing the dispersed phase to separate from the Y-shaped inlet of the micro-channel. With these two forces dominating the surface tension force, the dispersed phase becomes separated from the Y-junction and forms a clump. As the aqueous phase flows back into its designated entrance, the clump is completely detached and moves down the main channel. This process repeats in an alternating manner. The size of the clumps produced by the two-phase flow and the physical characteristics of the fluids used can be altered.

As the overall flow rate rises, slug flow transforms into either plug flow or droplet flow. The flow scheme depends on the form and organic phase rates. If the flow rate of the aqua phase is reduced while the organic phase rate is increased, the flow model will switch from slug flow to droplet flow, as shown in Fig. 3a with an aqua flow rate of 600 and organic flow rate of 30 µl per minute. If the aqua flow rate remains constant while the organic phase rate increases, the resulting flow model will always be plug flow, as illustrated in Fig. 4a with aqua and organic flow rates both at 500 µl per minute. Figure 5a-c show the chloroform volume fraction slug flow, droplet flow and parallel flow, respectively.

**Figure 6:**
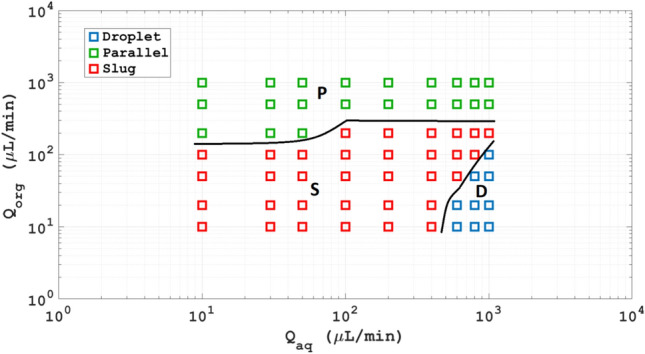
The flow models of chloroform-water based on computational work.

The flow maps in Fig. 6 demonstrate the impact of flow rate on flow regimes in a liquid–liquid system. At a moderate state, comparable flow rates for the aqua and organic phase, slug flow are observed. As the overall flow rate rises, the flow regime shifts to plug flow or droplet flow. If the aqua flow rate is maintained constant and the organic phase flow rate increases, the flow model transforms from slug to droplet or plug flow, depending on the type and flow rate of the organic phase. On the other hand, if the organic flow rate is increased whereas keeping the aqua flow rate uniform, the consequential flow model is permanent plug flow, as demonstrated in Fig. 6.

**Figure 7:**
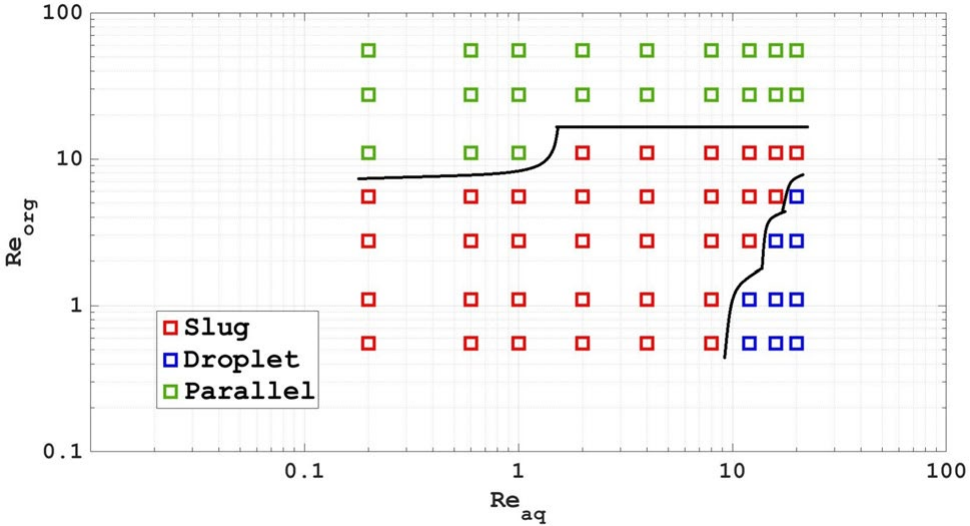
Flow pattern maps of the two-phase systems with Re as the coordinates based on computational work.

**Figure 8:**
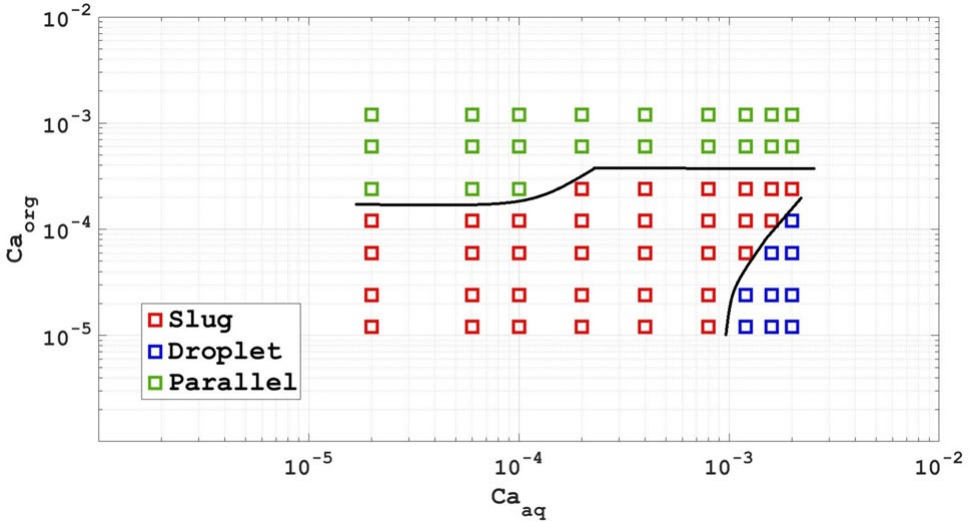
Flow pattern maps of the two-phase systems with Ca as the coordinates based on computational work.

**Figure 9:**
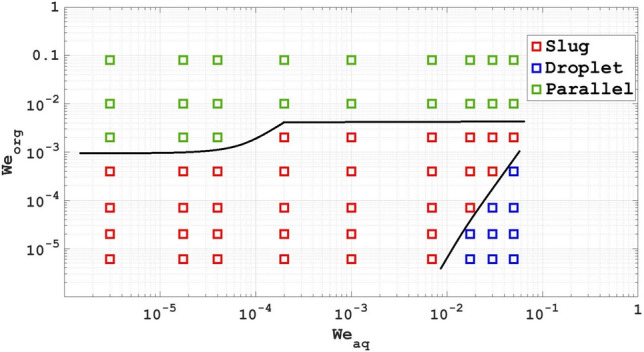
Flow pattern maps of the two-phase systems with We as the coordinates based on computational work.

Figures 7, 8 and 9 show flow pattern maps of the two-phase systems with Re, Ca and We numbers as the coordinates based on computational work, respectively. As can be seen from Fig. 7, at higher Re numbers of the organic phase, the flow moves towards the parallel flow. At high Re numbers of the aqueous phase and low Re numbers of the organic phase, the flow approaches are droplet flow. At equal Re numbers as well as low Re numbers, we will have slug flow.

Figures 8 and 9 of the diagram are similar to what happened for the Re number, with the difference that in Fig. 9, the range of the Weber number covers a larger range than the two dimensionless numbers Re and Ca.

## Conclusions

In this paper, a new model was presented, based on which the flow pattern in the serpentine microchannel can be predicted. In addition, this research examines the flow behavior of liquid–liquid extraction using chloroform and water in a serpentine microchannel. A 3D model was employed to calculate the flow behavior in the serpentine microchannel, and the results were compatible with experimental data. The impact of the flow rate of chloroform and water on the flow pattern was analyzed, revealing that slug flow occurs at low and comparable flow rates, but transitions to parallel or droplet flow as the total flow rate improves. Increasing the aqua flow rate while maintaining the organic phase flow rate stable lead to a shift from slug flow to either droplet flow or plug flow. The flow patterns in the serpentine microchannel were also depicted. In addition, flow pattern maps of the two-phase systems with Re, Ca and We numbers as the coordinates based on computational work are present. Resuts show that at higher Re, Ca and We numbers of the organic phase, the flow moves towards the parallel flow. At high Re, Ca and We numbers of the aqueous phase and low Re, Ca and We numbers of the organic phase, the flow approaches are droplet flow.

## Data Availability

The datasets used and/or analyzed during the current study are available from the corresponding author on reasonable request.
